# Leveraging the Single-Session Intervention approach to address psychological distress following a traumatic event: Opportunities to advance early intervention for trauma

**DOI:** 10.1371/journal.pmen.0000618

**Published:** 2026-06-08

**Authors:** Kathryn O’Neill, Anushka Patel, Avia Gray, Laura Bond, Claire Bird, Mercedes Aguerrebere, Marisol Vargas Vilugron, Denise Hernandez, Anne Marie Warren, Vikram Patel, Katherine Sanchez, John Naslund

**Affiliations:** 1 Department of Global Health and Social Medicine, Harvard Medical School, Boston, Massachusetts, United States of America; 2 Center for Digital Mental Health, Massachusetts General Hospital, Boston, Massachusetts, United States of America; 3 Patient and Community Engaged Research (PACER) Center, Baylor Scott & White Research Institute, Dallas, Texas, United States of America; 4 Baylor Scott & White Medical Center, Dallas, Texas, United States of America; 5 Trauma Research Center, Baylor Scott & White Research Institute, Dallas, Texas, United States of America; PLOS: Public Library of Science, UNITED KINGDOM OF GREAT BRITAIN AND NORTHERN IRELAND

## Abstract

Traumatic events are commonly experienced in the general population. Among those exposed, trauma-related mental health conditions, such as PTSD, depression and anxiety disorders, represent a significant cause of disability and impose considerable burden on affected individuals and their families. The symptoms of these mental health conditions can occur following traumatic events such as intimate partner violence, child abuse, and neighborhood violence, as well as in the aftermath of large-scale traumatic events such as natural disasters or mass shootings. Persistent symptoms of traumatic stress that go unaddressed can increase the risk of development of stress-related disorders and further disability, underscoring the need for scalable, accessible interventions. In this commentary, we explored the current literature on single-session interventions (SSIs) and brief early psychological interventions for trauma-exposed populations to reflect on opportunities to adapt these scalable single session interventions as a first step in the continuum of care to support early intervention and response to traumatic stress. Guided by the UK Medical Research Council and NIH frameworks for complex interventions, we synthesized the evidence from six systematic reviews and one umbrella review on single-session interventions (SSIs) for various mental health conditions and nine systematic reviews on brief and early psychosocial interventions for trauma. Findings suggest that trauma-informed, phase-specific, and ethically delivered SSIs, such as those incorporating active skill-building from cognitive behavioral therapy, may have potential as low-intensity, scalable entry points within a broader continuum of care. Overall, the SSI approach may offer promise for improving mental health outcomes after traumatic experiences. Further research is needed to optimize the effectiveness of these approaches, as well as the long-term impact, implementation, and delivery in underserved community settings where the need for such interventions is great.

## Introduction

In the United States, over 80% of individuals will be exposed to a traumatic event at least once in their lifetime and at least 8% of those individuals will develop subsequent PTSD [1,2]. Additionally, prevalence estimates for major depressive disorder following a large-scale traumatic event in a community range from 5-10% [3]. Inadequately treated or untreated stress-related disorders have far reaching consequences including loss of work productivity, unemployment, homelessness, domestic violence, and suicide [4]. Trauma-related symptoms are more even common after exposure to interpersonal violence, such as intimate partner violence, child abuse, and neighborhood violence. A global survey assessing lifetime trauma exposure and PTSD prevalence found the most common traumatic events for individuals were largely interpersonal such as the unexpected death of a loved one (31.4% of survey respondents), physical violence in childhood or as an adult (22.9%), and intimate partner or sexual violence (14.0%) [5]. People who have been exposed to interpersonal violence compared to large-scale traumatic events were found to have the highest risk of subsequent PTSD [5]. Additionally, the increasing frequency and severity of natural disasters, as well as violent events in the United States such as mass shootings, represent important contributors to stress-related disorders in the population [4,6]. For instance, a study evaluating the effect of 2017 Hurricane Maria on PTSD among Puerto Ricans who were displaced and moved to Florida after the storm versus those who remained on the island, found that there was an increased prevalence of anxiety, depression and PTSD in both groups compared to rates prior to Hurricane Maria [7]. Another study examining the mental health of witnesses and survivors of the mass violence incident at the 2017 Harvest Music Festival in Las Vegas, Nevada, found that four years after the event nearly half of participants (49.2%) experienced a major depressive episode and 63.3% experienced PTSD symptoms in the year prior to the survey [8]. Given the detrimental impacts of trauma for individuals and their communities, there is critical need for scalable interventions to support early response and promote recovery following experiences of trauma and in the aftermath of large-scale traumatic events. Early intervention approaches hold promise for preventing the development of stress related and other mental health disorders.

Despite this need, the development of single-session psychological interventions for trauma-related outcomes has been limited. Hesitancy to pursue single-session approaches for trauma may stem from several well-founded concerns. First, PTSD is a heterogeneous and multifaceted disorder, encompassing intrusive symptoms, avoidance, cognitive and emotional dysregulation, and functional impairment, with wide variability in symptom presentation and frequent comorbidities such as depression and substance use disorders [9,10]. This clinical complexity, shaped by individual risk factors and environmental contexts, has historically reinforced the assumption that sustained, multi-session interventions are necessary for effective treatment [11]. Second, trauma recovery is inherently nonlinear. Longitudinal research demonstrates multiple post-trauma trajectories, including resilience, normative recovery, delayed symptom onset, and chronic distress [12]. This variability complicates the identification of a single intervention timing or approach that would be universally appropriate following trauma exposure. Third, the field has been shaped by cautionary experiences with poorly implemented early interventions, most notably critical incident stress debriefing (CISD), a single-session intervention that was widely disseminated but later found to be ineffective or harmful in some cases [13,14]. These findings underscored the risks of premature emotional processing, lack of individual readiness, and insufficient attention to cultural and contextual factors, contributing to enduring skepticism toward brief trauma-focused interventions [15].

Psychiatric and psychological services provided in response to traumatic experiences are most effective when they are simple, brief, immediate, and practical [16]. In contrast to earlier approaches such as CISD, contemporary single-session interventions (SSIs) emphasize ethical delivery, voluntary participation, and alignment with an individual’s symptom profile, readiness, and contextual needs. SSIs are structured programs intentionally designed to involve only one encounter with a provider and typically distill core mechanisms from evidence-based psychotherapies, such as problem-solving or coping skills [15,17]. SSIs may function as stand-alone supports or as an initial step within a broader continuum of care, and their low resource requirements make them highly scalable [15,17]. Non-specialist or lay providers, ranging from dental hygienists to peer specialists, have demonstrated effectiveness in delivering SSIs for various mental health conditions [18]. A 2017 meta-analysis found self-administered SSIs to be no less effective than SSIs administered by therapists [19]. SSIs represent a promising approach to address the need-access gap for a variety of mental health conditions and situations because they offer flexibility in implementation and they can be self-administered or a provider, delivered online or in person, and implemented in various settings (e.g., hospitals, clinics, community organizations, and schools) [15,17]. Trials of conventional interventions consisting of multiple sessions to prevent the onset of stress related disorders following exposure to a traumatic event(s) face challenges such as low engagement and attrition [20]. A single session approach could serve as a preliminary intervention for trauma exposed individuals and offer an interim solution to the challenge of high drop-out rates of many multi-session interventions.

Over the last decade there has been mounting evidence demonstrating the acceptability, feasibility and efficacy of SSIs [18]. SSIs have shown effectiveness in improving individual-level outcomes for numerous mental health conditions including: alcohol use disorder, disordered eating, anxiety, depression, and self-harming behaviors [15]. A recent umbrella review found that of 24 systematic reviews identified, 20 reported beneficial effects of SSIs on one or more mental health or service engagement outcomes [18]. Two of the remaining reviews reported that SSIs did not outperform usual care and the other two were inconclusive due to limited number of studies [18]. A meta-analysis of studies using an SSI version of Acceptance and Commitment Therapy (ACT) - a proven psychological transdiagnostic intervention for supporting individuals to accept aversive psychological experiences and painful realities while guiding committed action towards personal values - for patients with chronic health conditions delivered in a mix of clinical and nonclinical settings, found generally positive results favoring SSI ACT and demonstrating that the intervention was acceptable and feasible [21].

Although many studies support the efficacy and scalability of SSIs, there has been little attention to leveraging this approach to respond to exposure to traumatic events as a means to prevent the development of stress related disorders and other mental health conditions or reduce or alleviate acute distress in community-based settings. Therefore, by reviewing and reflecting on the existing literature in this commentary examining published systematic and umbrella reviews, we offer insights for guiding the development and use of SSIs for early intervention in response to both interpersonal and community trauma exposure in a conceptual commentary on implications for practice. The objectives in this commentary were threefold: 1) to summarize the evidence on the impact of SSIs and their key components; 2) to summarize the evidence on brief psychological early interventions for responding to distress following a traumatic event; and 3) to discuss the potential for SSIs to address psychological distress in response to exposure to traumatic events.

### Ethics statement

This commentary includes only prior published data and had no human subjects. This commentary was exempt from Institutional Review Board review.

## Methods

In this commentary, we followed the National Institute of Health Research and UK Medical Research Council’s framework for complex interventions [22]. In aligning with this framework, our commentary involved summarizing the relevant evidence and initial formation of a conceptual model [22]. Our intention was to provide a high-level synthesis of the evidence base for SSIs and early brief interventions for trauma from prior published literature reviews. We opted for this broad approach given that our goal was to inform the immediate next steps for developing an SSI tailored to responding to exposure to trauma and delivered by non-specialist providers in under-resourced settings to prevent the onset of more serious stress-related or mood disorders. A formal systematic review would not have been appropriate for the current project given that our goal was not to provide an exhaustive summary of the literature. Rather, our aim was to reflect on the findings from the included reviews and discuss opportunities to apply these lessons to future intervention development.

We conducted two searches of PubMed: first, for literature reviews of SSIs and their efficacy using search terms relevant to “single session intervention;” and second, concurrently, for literature reviews of evidence-based interventions to address psychological stress following exposure to trauma using search terms relevant to “brief trauma interventions.” We included only reviews specifically mentioning SSIs or brief interventions (defined as being less than 3 months and fewer than 8 sessions). In addition, we discuss Psychological First Aid (PFA) as an illustrative case: PFA is a brief intervention designed for the acute phase (i.e., within minutes or hours of event exposure). While PFA was not explicitly part of our primary search but as it is single session in format, we lean on a separate integrative review (see Ling et al., 2025 for a detailed review). The research team reviewed the selected systematic and umbrella reviews to identify key components and themes of successful SSIs and brief interventions for trauma and summarized the details from these studies in Tables 1 and 2, respectively. Data extracted from the systematic reviews included: target populations, target mental health conditions, interventions, intervention providers, and intervention settings.

**Table 1:** Summary of the evidence on Single-Session Intervention approach.

First author and year	Included studies	Countries	Targeted Mental Health Condition	Target Population	Psychosocial interventions	Controls	Intervention providers	Intervention settings
Bertuzzi, Vanessa (2021)	18 RCTs	Australia, Iran, Netherlands, Norway, Romania, Sweden, Switzerland, United Kingdom, United States	Anxiety disorders	Youth and adults	CBT, parent-augmented one session treatment, cognitive restructuring intervention, culturally adapted one session treatment, imagery rescripting, virtual reality	Educational support therapy, waiting list, CBT, information only, self help, cognitive restructuring	Clinical psychologists, graduate students, dentists and dental hygienists, self-administered	Therapist offices, dental clinics, college campuses, emergency departments, outpatient clinics, on public buses
Bosse Chartier, Gabrielle (2023)	14 articles from 8 distinct studies	Finland, United Kingdom, United States	Suicide-related thoughts or behaviors	Patients in the emergency department (majority of studies was either adolescent or veteran populations)	Crisis Response Plan, Safety Planning Intervention + , Specialized Emergency Room Program, Family Intervention for Suicide Prevention, Family Based Crisis Intervention, Therapeutic Assessment, none	Contract for safety, care as usual	Clinicians	Emergency departments
Dochat, Cara (2021)	14 articles from 13 distinct studies	United States	Anxiety, depression, PTSD, or general mental health	Patients with chronic health conditions	Single session Acceptance and Commitment Therapy	Diabetes management education, waitlist, care as usual, enhanced care, support and migraine education	Doctoral students, clinical psychologists, neurologists	Community health centers
Kim, Jongtae (2023)	6 RCTs	Australia, Colombia, United Kingdom, United States	Common mental disorders	College students, family/non-family carers, counseling center attendees, general adults	Behavioral activation, dialectical behavior therapy, solution-focused psychotherapy	Waitlist, no treatment, relaxation training, problem-focused psychotherapy	Clinical psychologists, graduate students	Outpatient clinics
Rose, Suzanna (2003)	11 RCTs	Australia, Ireland, United Kingdom	PTSD prevention	Motor vehicle accident victims, relatives of seriously ill/injured, experienced miscarriage, assault or dog bite victims, acute burn trauma victims, mothers following childbirth, accident and emergency attenders, victims of violence	Immediate review & 3 month social worker input, individual counseling, debriefing, advice and leaflet, interactive interview	Care as usual, advice and leaflet	Therapists	Hospitals
Schleider, Jessica (2025)	24 systematic reviews consisting of 415 NRTs and RCTs	Australia, Belgium, Brazil, Canada, China, Colombia, Denmark, Finland, Germany, India, Iran, Israel, Japan, Malaysia, Netherlands, New Zealand, Norway, Romania, Spain, Sweden, Switzerland, Taiwan, Turkey, United Kingdom, United States	Substance use, anxiety, depression, eating problems, externalizing problems, general functioning or multiple mental health problems, suicide-related thoughts and behaviors, service use and engagement	Youth and adults	In-vivo exposure, behavioral activation, growth mindset, solution-focused therapy, dissonance-based interventions, brief behavioral parent training, exercise-based intervention, safety planning, contract for safety, family-based narrative intervention, motivational interviewing, brief personalized feedback	Care as usual, waitlist, placebo, multisession therapy, assessment, no control	Self-administered, mental health professionals, dental hygienists, emergency department nurses, peer specialists, trained research staff, graduate students, social workers, physicians, music therapists	Primary care clinics, schools and universities, emergency departments, general dentistry practices, online
van Emmerik, Arnold (2002)	5 RCTs 1 NRT 1 CCT	United Kingdom	PTSD prevention	Victims of motor vehicle accidents, violent crime, or burns; police officers; women who’ve had a miscarriage; individuals exposed to combat	Critical incident stress debriefing, historical group debriefing	No intervention control	Nurses, psychiatrist, police officers, social workers	Outpatient clinics, police departments

**Table 2:** Summary of the evidence on brief interventions to address psychological distress following a traumatic event.

First author and year	Included studies	Countries	Target populations	Psychosocial interventions	Controls	Intervention providers	Intervention settings
Birur, Badari (2017)	34 RCTs 14 observational studies 4 others (pilot, quasi experimental)	Australia, Canada, Germany, Israel, Netherlands, United Kingdom, United States	Victims of motor vehicle accidents, burns, industrial accidents, sexual assault, nonsexual assault, or terrorist attacks; mothers of preterm infants or experienced traumatic births; diagnosed with acute stress disorder; physical injury; patients who received major surgery; exposed to combat or earthquake	Trauma-focused CBT, supportive counseling, psychological debriefing, caregiver-child support, collaborative care, writing intervention, self-help booklet	Assessment only, supportive counseling, wait-list, standard care, placebo, SSRI, self-help booklet, psychoeducation	Self-guided, clinical psychologists, nurses, psychiatrists, social workers, graduate students, case managers, trauma support specialists, trained study staff, teachers	In patient units, outpatient clinics, telephone, online, participant’s homes, schools
Bisson, Jonathan (2021)	69 post-incident RCTs 6 pre-incident RCTs	Australia, Canada, China Denmark, France, Germany, Hong Kong, Ireland, Israel, Japan, Mexico, Netherlands, Sweden, Switzerland, United Kingdom, United States	Victims of motor vehicle accidents, violent crime, workplace violence, burns or armed robbery; women who experienced miscarriage or emergency cesarean section; parents of children with a cancer diagnosis; fire and emergency service workers; military peacekeepers; exposed to combat, earthquake or life-threatening experience; physical injury and patients with a life-threatening diagnosis	Stress inoculation training, heart rate variability cognitive bias feedback, MPAS resilience training, attention bias modification training, psychological debriefing, computerized visuospatial task, EMDR, psychoeducation, counselling, perinatal parenting intervention, trauma processing therapy, CBT, communication facilitator, IPT, intensive care recovery program, collaborative care, structured writing therapy	Waitlist, treatment as usual, symptom monitoring, repeated assessment, other minimal-attention group, an alternative psychological treament	Nurses, trained study staff, self-administered, trained military personnel, psychiatrists, clinical psychologists, social workers, peer supporter, medical or doctoral students, midwives, trauma support specialists, case managers	Military bases, online, outpatient clinics, in patient units, fire departments, participant’s homes, retail stores, telephone, factories
Bryant, Richard (2007)	11 RCTs 3 long-term follow up 1 clinical controlled trial 1 meta-analysis	Australia, Ireland, Israel, United Kingdom, United States	Victims of crime, burns, motor vehicle accidents, sexual assault, nonsexual assault, or industrial accidents; emergency room patients	Psychological debriefing, CBT	Assessment only, supportive counseling, standard care, supportive listening, symptom monitoring	Clinical psychologists, trained research staff, midwives, nurses, psychiatrists	Businesses, military, schools, in patient units, outpatient clinics, emergency departments, telephone
Daniel, Nadia (2024)	31 RCTs 6 clinical controlled trials	Australia, Austria, Finland, Greece, Germany, Israel, Italy, Jordan, Lebanon, Malaysia, Netherlands, Norway, South Korea, Sri Lanka, Switzerland, Tanzania, Thailand, Turkey, Uganda, United Kingdom, United States, Western European countries	Refugees, asylum seekers and internally displaced people	CBT-based brief interventions	Waitlist, care as usual, enhanced care as usual, no-treatment monitoring group, meditation-relaxation techniques	Trained peer refugees, trained facilitators from local community, clinical psychologists, psychiatrists, social workers, graduate students, mental health professionals, trained lay counsellors	Non-governmental organizations, participant’s homes, refugee camps, outpatient clinics, child and adolescent welfare programs, schools
Howlett, Jonathan (2016)	11 RCTs 7 systematic reviews 1 latent class analysis 1 meta-analysis	Israel, United Kingdom, United States	Victims of violent crime, motor vehicle accidents, or sexual assault; military members; emergency service workers	Psychological debriefing, CBT, collaborative care, memory structuring intervention, psychoeducation, informational videos, Child and Family Traumatic Stress Intervention	Waiting list, antidepressant medication, assessment only, usual care, supportive listening	Clinical psychologists, social workers, nurse practitioners, nurses, self-administered	Outpatient clinics, in patient units, telephone, military bases
Kliem, Soren (2013)	13 RCTs	Australia, Germany, Israel, Netherlands, United Kingdom, United States	Victims of motor vehicle accidents, nonsexual assault, industrial accidents, sexual assault, or terrorist attacks	Trauma-focused CBT	Supportive counseling, cognitive restructuring, standard care, waitlist, assessment only	Clinical psychologists	Outpatient clinics and emergency departments
Kornor, Hege (2008)	7 articles from 5 RCTs	Australia, United States	Victims of motor vehicle accidents, industrial accidents, sexual assault or nonsexual assault; diagnosed with acute stress disorder	Trauma-focused CBT	Supportive counseling	Clincial psychologists	Outpatient units
Roberts, Neil P (2010)	15 RCTs	Australia, Netherlands, Spain, United Kingdom, United States	Victims of motor vehicle accidents, industrial accidents, nonsexual assault, sexual assault, occupational injury, or violent crime; physical injury; civilians exposed to traumatic events	CBT, supportive counseling, cognitive restructuring, behavioral activation, collaborative care, writing intervention	Waitlist, supportive counseling, minimal intervention, standard care, information only, progressive muscle relaxation training, self-help booklet, structured writing intervention	Clinical psychologists, trained research staff, case managers, trauma support specialists, psychiatrists	Emergency departments, outpatient clinics, telephone
Roberts, Neil P (2019)	27 RCTs	Australia, Canada, China, Denmark, France, Iran, Israel, Italy, Portugal, Netherlands, Norway, Sri Lanka, Sweden, Switzerland, United Kingdom, United States	Parents whose children are discharged from the PICU or have children diagnosed with cancer; victims of aggression, motor vehicle accidents, armed robbery or life-threatening events; mothers of preterm infants or experienced traumatic births; Patients in the ICU, who’ve received mechanical ventilation or a transplant or had a physical injury	Psychoeducational tool, CBT, psychological first aid, parenting intervention, individual and group counseling, communication facilitators, memory structuring intervention, intensive care recovery program, intensive care diaries, parent child intervention, psychological debriefing, consultation with a midwife, creative arts	Waitlist, care as usual, supportive listening, education program, parenting support, parent targeted intervention (massage, relaxation and imagery), psychological first aid, assessment only	Self-administered, nurses, psychologists, psychiatrists, physicians, midwives, social workers, trained study staff, artists, trauma support specialists, communication facilitators, trained counselors, healthcare staff, graduate students, psychologically trained artist	In patient units, outpatient clinics, telephone, communities, military bases, major convienience store chain

We then used the AMSTAR-2 (A MeaSurement Tool to Assess systematic Reviews) instrument to assess the quality of the included systematic reviews [23]. The AMSTAR-2 utilizes 7 domains made up of 16 items to assess the overall risk of bias of each review [23]. The domains include registration of the protocol before the start of the review, adequacy of the literature search, justification for excluding individual studies, risk of bias of individual studies assessed, appropriateness of meta-analytical methods, consideration of risk of bias when interpreting the results of the review, and assessment of presentation and likely impact of publication bias [23]. The AMSTAR-2 quality assessment was done by one member of the research team (KO). Throughout the data extraction and quality assessment, the research team met regularly and reviewed uncertainty to reach consensus.

## Results

We identified 6 systematic reviews and 1 umbrella review investigating the effectiveness of SSIs (Table 1), of which 3 included a meta-analysis. The sum of clinical trials reported across the reviews was 478. Due to the related nature of many of the reviews, there was likely overlap in the included trials among some of the reviews. We also identified 9 systematic reviews investigating brief early interventions to prevent the onset of PTSD and other mood disorders following trauma exposure (Table 2), of which 3 included a meta-analysis. The reviews of brief and early interventions for trauma examined the results of 236 clinical trials collectively. Since the reviews had similar research questions, it is possible that there is overlap among the included clinical trials in some of the reviews. Two reviews focusing on SSIs examined the effectiveness of an SSI for PTSD prevention and psychological debriefing. Both reviews indicated there was little to no evidence that SSI psychological debriefing was effective in preventing PTSD. Trauma-focused cognitive-behavioral therapy (CBT) consistently demonstrated the strongest evidence for PTSD prevention, particularly in individuals exhibiting early trauma symptoms (Table 2).

### Quality assessment

Four of the systematic reviews of SSIs for various mental health conditions were determined to have high methodological quality while two reviews had moderate methodological quality and one was of low methodological quality. Three of the systematic reviews of brief and early interventions for trauma had high methodological quality, while three systematic reviews had moderate methodological quality, and three of the systematic reviews had low methodological quality. The methodological quality ratings for the included reviews are summarized in S1 Table.

## Objective 1: Summary of the evidence on single-session intervention approach

### Review characteristics

The included reviews explored the efficacy of SSIs across various mental health conditions and populations. Two reviews included trials of interventions to treat common mental disorders [21,24], two reviews focused on PTSD prevention [25,26], one review focused on anxiety disorders [27], one focused on suicide-related thoughts and behaviors [28], and the umbrella review [18] covered interventions for a wide variety of mental health conditions. Four reviews focused on interventions exclusively in adult populations [21,24–26] and the remaining three included trials with both adult and youth participants [18,27,28]. SSIs were most frequently delivered in clinical settings by clinicians and trained mental health professionals. However, a small number of the SSIs were self-administered or delivered by lay counselors without formal mental health care training. Overall, the SSIs included adapted cognitive behavioral therapy (such as behavioral activation or problem solving), exposure-based therapies, psychological debriefing, parenting interventions, and motivational interviewing. The interventions are summarized in Table 1.

### Effectiveness of SSIs

Schleider et al. have conducted multiple reviews on SSIs in youth and adults, demonstrating positive outcomes across multiple mental health concerns [18,19]. Their recent umbrella review of SSIs spanning multiple mental health conditions, including substance use, anxiety, depression, eating disorders, externalizing behaviors, suicide-related thoughts and behaviors, and overall mental health functioning synthesized the findings from 24 systematic reviews, encompassing 415 distinct trials [18]. The review reported generally positive effects for SSIs across different conditions and populations. However, studies with shorter follow-up periods showed greater success, suggesting that SSIs may work best as supplementary treatments rather than standalone interventions [18]. The research also highlighted the range of approaches for delivering SSIs, ranging from self-administered to those delivered by mental health professionals, emergency department nurses, and even dental hygienists [18].

### SSIs for common mental disorders

The two systematic reviews of SSIs for common mental health disorders reported positives effects but raised caution in interpreting the results due to lack of statistical significance or methodological bias in the included trials [21,24]. A systematic review and meta-analysis examining the use of Acceptance and Commitment Therapy (ACT) in an SSI format for individuals with chronic health conditions experiencing anxiety, depression, PTSD, or general mental health difficulties suggested moderate positive effects of ACT SSI on functioning and well-being, though this was not statistically significant [21]. Among the 5 studies included in the meta-analysis conducted by Dochat et al. (2021), the reported effect sizes came from two pilot trials with small sample sizes, meaning the results of the overall meta-analysis should be cautiously interpreted [21]. While SSIs showed preliminary feasibility and acceptability, there is a need for larger, more rigorous studies to establish their efficacy [21]. Similarly, another systematic review of SSIs for common mental disorders in adults noted positive effects, particularly for depression, but warned against high bias in study methodologies and suggested that any results should be interpreted tentatively [24]. The interventions in this review included behavioral activation, dialectical behavior therapy (DBT), and solution-focused psychotherapy, delivered by trained mental health professionals [24]. Again, while SSIs showed promise in reducing depressive symptoms, their effectiveness for anxiety was less consistent [24]. Both of the reviews were assessed as having high methodological quality using the AMSTAR-2 assessment [21,24].

### SSIs for anxiety disorders

Two reviews evaluated SSIs specifically for anxiety disorders and found preliminary effectiveness in reducing anxiety symptoms [21,27]. In the systematic review by Bertuzzi et al (2021), the efficacy of SSIs for anxiety disorders was investigated in both youth and adults, with interventions including cognitive-behavioral therapy (CBT)-based exposure techniques, parent-augmented treatments, cognitive restructuring, culturally adapted therapies, and virtual reality exposure [27]. SSIs for anxiety disorders demonstrated effectiveness in improving cognitive, behavioral, and physiological outcomes. SSIs were superior compared to no treatment control conditions in reducing anxiety symptoms, and no differences in effectiveness were observed when compared to multi-session treatments [27]. The authors of this review suggest that SSIs may have a powerful role in empowering individuals to manage their anxiety symptoms independently but also highlighted the need for further research into their long-term cost-benefit balance [27]. A systematic review evaluating SSIs based on ACT for patients with chronic health conditions, found that SSI ACT was effective at reducing anxiety symptoms in all trials which included anxiety symptoms as an outcome except for one trial with breast cancer patients [21]. One of the trials included in the review found that the skill of psychological flexibility taught in the ACT SSI reduced anxiety and depression symptoms in those at risk for cardiovascular disease [21]. Both of the reviews were assessed as having high methodological quality using the AMSTAR-2 assessment [21,27].

### SSIs for PTSD prevention

Two systematic reviews assessed the effectiveness of single session psychological debriefing in preventing PTSD among trauma-exposed individuals [25,26]. There was some overlap between the reviews, with five studies included in both, resulting in a total of 13 unique trials [25,26]. Neither review found evidence to support the effectiveness of a single session psychological debriefing intervention to prevent the onset of PTSD [25,26]. In the review by Rose et al (2003), of the 11 trials involving diverse experiences of trauma, including motor vehicle accident survivors, survivors of violence, and women who had experienced miscarriage or childbirth trauma, 6 trials had neutral outcomes and 2 showed negative effects of the SSI [25]. Studies that had the longest follow up period were more likely to show negative effects [25]. In the review by van Emmerik et al (2002), seven trials of single-session psychological debriefing interventions were evaluated, and no clear evidence emerged that these SSIs could prevent PTSD [26]. A subset of the studies indicated that the intervention may interfere with the natural processing of a traumatic event and may be harmful to patients [26]. It should be noted that one of these reviews as assessed has having moderate methodological quality [25] and the other was assess as having low methodological quality [26] using the AMSTAR-2 quality assessment.

### SSIs for suicide-related thoughts and behaviors

One review focused on SSIs designed for individuals experiencing suicide-related thoughts or behaviors, specifically those receiving emergency room care [28]. The review analyzed crisis-focused interventions such as safety planning, crisis response plans, and family-based crisis interventions delivered by clinicians in emergency settings. These interventions improved patient engagement with follow-up care, though their impact on immediate symptom reduction was mixed due to variability in study outcomes. A limitation with the studies included in the review was the narrow focus on adolescent and veteran populations, highlighting the need for research on broader population groups. This review [28] was assessed has having moderate methodological quality using the AMSTAR-2 quality assessment.

## Objective 2: Summary of the evidence on brief interventions to address psychological distress following a traumatic event

### Review characteristics

There were nine systematic reviews assessing studies of brief early interventions to address psychological distress following a traumatic event and the onset of PTSD and other mood disorders. In the studies summarized in each of the reviews, interventions were delivered to individuals with a variety of different trauma exposures including but not limited to motor vehicle accidents, earthquakes, sexual or nonsexual assault, or traumatic birth. The populations in these reviews were largely exposed to single-incident events and were not exposed to chronic trauma. The interventions were delivered across a mix of community and clinical settings by a variety of providers including trained peers, frontline healthcare workers, and other clinicians or were entirely self-guided. The specific interventions included in the reviews are summarized in Table 2, and consisted of trauma-focused cognitive behavioral therapy, exposure-based therapies, psychological debriefing, eye movement desensitization and reprocessing (EMDR), psychoeducation and motivational interviewing.

### Trauma-focused cognitive behavioral therapy

In all nine reviews, CBT or trauma-focused CBT emerged as having the most studies supporting intervention effectiveness for preventing PTSD following trauma exposure across various populations and settings [20,29–36]. CBT and trauma-focused CBT are structured goal oriented interventions aimed at identifying and changing harmful patterns of thoughts or behaviors [31]. Trauma-focused CBT uses the principles and techniques of CBT, which can be used to address a variety of mental health conditions, and applies them specifically to an individual’s reaction to trauma and trauma response [37]. All nine reviews indicated that CBT or trauma-focused CBT had the strongest effect sizes compared to both active comparator groups and treatment as usual or waitlist controls [20,29–36]. One comprehensive review of 52 trials aimed at individuals exposed to diverse traumatic events, including motor vehicle accidents, sexual assault, terrorist attacks, combat trauma, and surgical trauma found that the most effective interventions for acute stress disorder and PTSD were trauma-focused CBT and modified prolonged exposure therapy when compared to supportive counseling, wait list, standard care, escitalopram or placebo control conditions [20]. One review and meta-analysis assessing 6 pre- and 69 post-incident interventions for trauma-exposed individuals demonstrated effectiveness of trauma-focused CBT for individuals diagnosed with acute stress disorder [29]. However, the overall quality of evidence was low, and this review emphasized the need for further research to integrate interventions into healthcare systems effectively [29]. A review evaluating interventions to reduce PTSD onset in individuals with an acute stress disorder suggested that CBT-based strategies demonstrated preliminarily effectiveness [30]. The review also emphasized the role of cognitive trauma processing in determining PTSD development in trauma exposed individuals [30]. Another review included 34 studies of CBT interventions for refugees, asylum seekers and internally displaced people and demonstrated short term effectiveness in improving depression, anxiety and PTSD symptoms [31]. However, the effects dissipated at longer term follow-up [31]. The review also found no difference in effectiveness when comparing interventions delivered by clinicians and lay people [31]. Although the majority of the trials included in these reviews involved delivery of the interventions in person or over the telephone, one trial of internet-based CBT showed superior outcomes when compared to no treatment or treatment as usual control conditions [29]. The included reviews were a mix of high, moderate and low methodological quality as assessed by the AMSTAR-2 [20,29–36].

### Modified prolonged exposure therapy

Prolonged exposure interventions seek to modify the memory of a traumatic event before memory consolidation occurs and PTSD can subsequently develop [20]. Modified prolonged exposure therapy demonstrated preliminary effectiveness in the included reviews [20,29]. In a review by Bisson et al. (2021), a prolonged exposure-based intervention showed no difference in effectiveness compared to cognitive therapy in preventing the onset of PTSD [29]. Another review by Birur et al (2017) suggested that a single-session imaginal prolonged exposure intervention in emergency departments within the 24 hours following a traumatic exposure led to slight reductions in depression levels [38]. The intervention was 30–45 minutes and consisted of repeated therapist-guided imaginal exposure to the traumatic event, ongoing anxiety monitoring, and supportive processing. At the end of the intervention, the participant was given a behavioral homework assignment which included exposure to the trauma cue identified in the session [38]. However, the trial faced challenges such as small sample sizes, attrition, and reliance on waitlist control groups, limiting the generalizability of findings [20]. The review by Bisson et al. (2021) was assessed as having moderate methodological quality while the review by Birur et al. (2017) was assessed as having low methodological quality.

### Psychological debriefing

While psychological debriefing has been widely implemented, it lacks empirical support [30,36]. In a review of interventions for crime survivors, burn survivors, and assault survivors in various settings, including hospitals, businesses, and schools, psychological debriefing did not appear effective in preventing PTSD [30]. Another review also found no evidence supporting single-session psychological debriefing for PTSD prevention [36]. Both of these reviews were assessed as having low methodological quality using the AMSTAR-2 [30,36].

### Other interventions for trauma

A few additional interventions demonstrated preliminary effects in reducing PTSD symptoms and the onset of mood disorders following exposure to a traumatic event. A review of 61 studies covering diverse trauma-exposed populations, including military personnel, ICU patients, sexual assault survivors, and individuals affected by natural disasters found no significant differences between intervention and usual care when participants were not pre-screened for trauma symptoms [35]. However, for those exhibiting trauma symptoms, EMDR showed preliminary effectiveness in reducing PTSD symptoms [35]. This review was assessed as having high methodological quality [35]. Another review found the collaborative care approach offered benefits, though additional research is needed due to small sample size [36]. However, this review was assessed to have low methodological quality [36].

### Psychological first aid

Although not included in the systematic reviews summarized above, PFA provides an important contextual example of how a brief, acute-phase intervention has been conceptualized and implemented in the immediate moments and hours following a traumatic event. Psychological first aid is an intervention explicitly designed for delivery within the acute period of experiencing a traumatic stressor. It is meant to stabilize an individual who has experienced a trauma while still in crisis by restoring baseline functioning and facilitating adaptive survival and problem-solving skills to meet the moment. Although PFA was originally conceived during World War II, it has evolved in structure and clinical components over the decades [39] and is most often recommended following mass trauma events [40]. A recent integrative review identified 20 studies on PFA to examine its effectiveness, implementation, and experience on target populations [39]. This review found that PFA varies significantly in implementation regarding (i) timing of intervention, (ii) dosage (ranging from 1 session to 2 years of treatment), (ii) format (individual vs. group), (iii) delivery agents, and (iv) fidelity to the original protocol. Notably, PFA has been deployed with multiple populations outside the definitions of trauma survivors; examples include PFA deployment for adolescents experiencing bullying and people experiencing financial fraud [41,42]. Similarly, PFA is being deployed with police officers, who have routine trauma exposure of an expected kind, which is different from the original use in one-time mass trauma scenarios [43].

Overall, the various iterations of PFA make it difficult to compare across protocols, but the common elements observed across all PFA protocols included active listening, relaxation/stabilization, problem-solving/practical assistance, and social connection/referral. In terms of effectiveness, PFA reduced anxiety and facilitated adaptive functioning in the immediate aftermath of trauma. However, the evidence was less compelling for reducing PTSD and depression, which are downstream consequences after the initial shock and dysfunction of a traumatic exposure wears off.

## Objective 3: Clinical considerations for single-session interventions to address psychological distress following a traumatic event

Almost all of the SSIs reviewed in our study were designed and deployed in high-income and westernized cultural settings. Ample evidence now suggests that the clinical presentation of PTSD varies by cultural setting [44,45]. Yet, the lack of rigorous and systematic evaluations on brief interventions in general and SSIs in particular from low-and-middle-income countries is rather striking. Currently, there are very few SSIs designed and deployed for high-risk settings where humanitarian and natural disasters occur. Given the brevity and ease of implementing SSIs once providers are trained and that LMIC shoulder the lion’s share of natural disasters and humanitarian emergencies [46], this underscores a missed opportunity. In this next section, we provide clinical and implementation considerations to design SSIs, and these principles should be applied for underserved communities who have limited access to treatment resources.

### Designing SSIs to facilitate recovery after traumatic events: Clinical considerations

Ultimately, early interventions for trauma survivors (i.e., within the first three months of trauma exposure) are effective [47], but SSI formats have not gained traction since CISD. We discuss the factors that can influence the success of an SSI from a clinical lens. Given the heterogeneity in trauma responses [48], a ‘one-size-fits-all’ approach is not likely to work for SSI development. We consider the need for a suite of SSIs that serve as low-intensity ‘entry-points’ that speak to survivors’ chief presenting concerns.

**Fig 1:**
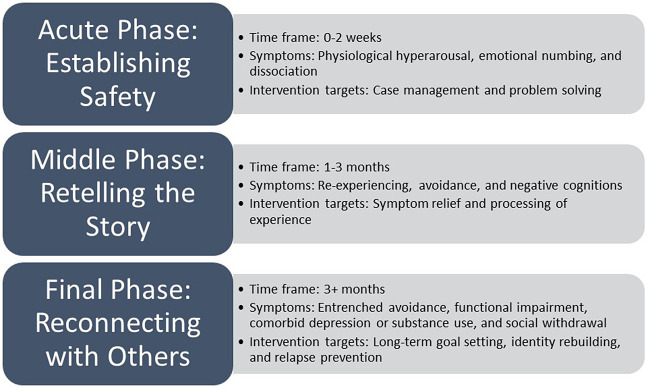
The temporal progression of trauma, its symptoms and intervention targets..

Understanding the temporal progression from trauma exposure to stress and trauma related disorders is crucial to determining when, how, and what kind of intervention is appropriate. Many cognitive-behavioral treatments for PTSD are premised on early theoretical and clinical observations that conceive of PTSD as a disorder of disempowerment and disconnection [49]. This foundation posits three phases of recovery: establishing safety, retelling the story of the traumatic event, and reconnecting with others and each phase has its own unique clinical needs [49]. Mapping these phases onto the current Diagnostic and Statistical Manual of Mental Disorders 5 Text Revision [50] criteria for PTSD, we conceptualize the first phase as the acute phase (0–2 weeks), where the clinical focus ought to be safety, stabilization, and re-establishing basic functioning. Interventions during this phase should focus on stress and anxiety management, case management, and problem solving [51]. One intervention with an evidence base that can be deployed for crisis management in this phase is PFA. We conceptualize PFA as an immediate *acute* crisis response tool rather than a recovery-oriented intervention. While the implementation of PFA has drifted in practice (Ling et al., 2025), its core techniques of anxiety management, linkage to resources, and problem-solving remain valuable tools for stabilizing a survivor in the minutes or hours after a traumatic event. As the acute stress responses subside, persistent symptoms such as re-experiencing, avoidance, and negative cognitions signal the development of PTSD during the middle phase of post-trauma adaptation (1–3 months). During this phase individuals need symptom relief and to process their experience; this phase is optimal for structured, trauma-focused interventions [47]. Some, but not all, individuals who have experienced trauma may experience the final phase which is chronic PTSD (3 + months) and is associated with the persistent middle phase symptoms leading to entrenched avoidance, functional impairment, comorbid depression or substance use, and social withdrawal; as such, treatment requires longer-term goal setting, identity rebuilding and relapse prevention [47]. Tailoring SSIs to these phases holds potential to ensure that interventions are not only safe but optimally effective for engaging a meaningful clinical target (Fig 1).

There are two primary strategies to developing a trauma-focused SSI: adapt an existing evidence-based protocol or design a treatment de novo (i.e., ‘from scratch’). We can create new SSIs or adapt existing evidence-based treatment using the BEST framework derived from basic research of social psychology [52]. The first element of the BEST framework is ‘*Brain science to normalize concepts in the program’*. Incorporating brain science-rooted explanations makes the take away messages of the SSI easier for participants to trust [52]. The second element is ‘*Empower individuals to a helper or expert role*’ [52]. Fostering a sense of self agency and centering the lived experience of individuals can increase buy-in from the target population for the intervention [52]. The third element is ‘*Saying is believing activities to solidify learning’*. ‘Saying-is-believing’ activities are intended to promote the internalization of new positive self-beliefs [52]. The final element is ‘*Testimonials and evidence from valued others’* which is thought to capture participants’ attention through engaging them cognitively and emotionally [52].

Selecting an appropriate clinical mechanism for an SSI is the first important step. While cognitive/behavioral avoidance is the primary mechanism that gold-standard PTSD treatments target, several additional clinical targets may be useful to consider. For example, shame and self-stigma are especially relevant in sexual assault and those experiencing moral injury [53–55]. Moral injury is defined as “perpetrating, failing to prevent, bearing witness to, or learning about acts that transgress deeply held moral beliefs and expectations” [56]. Healing from moral injury extends beyond symptom-level repair. Moral injury contributes to avoidance and social disconnection in a range of samples, and it has often been studied among those exposed to war [57]. Other clinical targets for SSIs that may yield promising results are guilt [58] and sleep-related problems such as insomnia and trauma-related nightmares [59,60].

SSIs with brief narrative or imagery-based interventions may help reconsolidate memory and reduce self-blame [61]. Guilt is a powerful driver of re-experiencing and withdrawal, particularly for those with combat-related PTSD. Brief writing or forgiveness exercises have shown promise in addressing guilt in some populations [62,63]. The last potential clinical target, nightmares, could be addressed through imagery rehearsal therapy [64]. There has been preliminary effectiveness in single session interventions in reducing trauma-related sleep disturbances [65]. These focal points offer efficient ways to interrupt PTSD maintenance mechanisms early.

### Delivering trauma-related SSIs: Implementation considerations

Even the best-designed SSI can fail or do harm if it is delivered without regard to trauma-informed principles [66]. According to US Substance Abuse and Mental Health Services Administration (SAMHSA), trauma-informed practice includes adherence to these principles: (1) safety – minimizing risk and building one’s sense of control; (2) transparency and trust – ensuring strategies and decisions are visible, described clearly, and do not violate trust in relationships; (3) peer support – voluntarily building mutual and respectful relationships; (4) collaboration and mutuality – rebalancing power differentials in decision making; (5) empowerment, voice and choice – acknowledging strengths and having space to use them; and (6) recognizing cultural, historical, and gender issues – avoiding stereotypes, promoting nurturing cultural practices and addressing historical trauma [67].

Trauma interventions should be delivered with attention to both emotional *and* physical safety before engaging in potentially triggering exercises [68]. To be clear, therapeutic content in SSI format should not be the focus of working with a trauma survivor unless physical safety is established. It is also critical that participants know what the intervention involves and be empowered to opt out. Participants should guide the session focus where possible, maintaining autonomy over pace and disclosure [67]. Including lay providers or peers in the delivery of SSIs can increase trust and cultural resonance of the intervention [67]. Additionally, SSIs must be adapted to the participants’ linguistic, spiritual, and social norms to ensure relevance and acceptability [67]. Lastly, when implementing an SSI, it is important to ensure there is appropriate institutional awareness of trauma informed care and that all points of contact, including waiting room staff in emergency rooms, are appropriately trained [67].

SSIs for use following trauma exposure are not inherently dangerous or ineffective; rather, their success hinges on several factors. Examples include 1) selecting clinical targets personalized to recovery phase, 2) creating an engaging design, 3) delivering care in an ethical and feasible format, and 4) aligning the care environment toward survivors’ clinical needs through trauma education. Learning from past mistakes, such as the case with CISD, the field should embrace a more nuanced and evidence-informed model that leverages SSIs as part of broader trauma response efforts. Trauma-informed SSIs hold potential for improving access to care when thoughtfully designed and deployed with rigorous evaluation procedures to ensure that any potential harms can be identified and addressed.

## Discussion

The summarized body of research on SSIs suggests their potential to mitigate various mental health conditions, though their effectiveness varies depending on the disorder, population, and treatment approach. In the majority of the trials across the included systematic reviews, SSIs were delivered by clinicians and trained mental health professionals [24,25,28], with relatively few trials of self-administered or peer-delivered SSIs [18]. Of the systematic reviews investigating brief trauma interventions, interventions were delivered in a mix of community and clinical settings and by a variety of provider types (e.g., trained peers, frontline health workers, clinicians) [20,29–36]. Further, methodological limitations, short follow-up periods, and mixed results across populations in the trials of SSIs included in the systematic reviews suggest that while SSIs are promising, at this stage, they are best used as supplements to standard care, thereby highlighting the need for more rigorous research to validate their long-term effectiveness. There remain two persistent gaps in the literature 1) a lack of evidence on SSIs to treat trauma-related symptoms and disorders and 2) a lack of evidence on SSIs for prevention of trauma-related disorders.

Across the nine systematic reviews of brief interventions for trauma included in this review [20,29–36], trauma-focused CBT consistently emerged as effective in preventing PTSD and reducing psychological distress following trauma exposure, suggesting that condensing trauma focused CBT into a single session or breaking it down into multiple single session interventions each targeted for different phases of trauma recovery could be a scalable solution to the prevention of PTSD and other stress related disorders. These CBT-based interventions, applied across diverse trauma types and settings, showed the strongest short-term effects in addressing traumatic stress when compared to other approaches such as supportive counseling, psychoeducation alone, or pharmacological controls [20,29–36]. The most effective SSIs for all target mental health conditions included not only brief psychoeducation but also included an active skill building component frequently used in traditional multi-session treatments such as healthy coping skills or behavioral activation [18]. SSIs offered in both community and clinical settings showed moderate effect sizes in improving mental health outcomes [18,21].

Although SSIs and early interventions may offer promising short-term benefits, their long-term effectiveness remains uncertain, emphasizing the need for further research on targeted approaches and implementation strategies [17,18]. Due to the dissipating long term effects, SSIs may prove most useful as a means of complementing, extending, and increasing the accessibility of existing mental health services, rather than replacing higher-intensity treatments [17,18]. Augmenting SSIs with a ‘booster’ session a few months following the initial treatment or with digitally designed adjuncts could potentially be an effective strategy to sustain the intervention’s initial effects [19]. Similarly, it is crucial to consider the clinical nuance of the index traumatic event and individual differences in response when deciding to deploy an SSI. For example, prolonged childhood abuse and neglect tends to elicit a complex PTSD response compared to single-event incidents and accidental events (e.g., natural disaster or car accident). Similarly, certain events – such as drug-facilitated sexual assault – can elicit high levels of shame and impair trauma memory to create dissociative responses whereas other traumatic exposures do not come with this presentation [69,70]. Ultimately, the range of traumatic events and individual responses – which are heterogenous in clinical presentation – are crucial nuances to consider when developing and deploying an SSI.

SSIs may provide an important early solution to reach individuals who cannot access or are hesitant to seek higher-intensity support and expand the reach of and impact of existing mental health services [18]. Integrating SSIs into existing care systems as a first line treatment may have potential to bridge gaps in support systems. When compared to control groups who do not receive an intervention, most SSIs show low to moderate effect sizes in symptom reduction for various mental health conditions [17]. Although SSIs may not be as effective as longer treatments for reducing mental health symptoms, they are more cost effective than multi-session treatments [17]. Meta-analysis has found self-administered SSIs were as effective as therapist lead SSIs for youth populations [19]. The option of providing SSIs as a self-administered intervention greatly reduces the monetary costs of providing the intervention without sacrificing effectiveness [19]. Collectively, these reviews suggest that while SSIs can provide accessible and immediate mental health support, their effectiveness varies by condition, population, and intervention type. Further research is needed to optimize their application, particularly in terms of long-term benefits and integration into broader care frameworks.

A continuum of care is imperative and should be the standard of care for mental health services following a traumatic event, the same way established continuums of care exist for physical health services [16]. A growing body of research has explored the efficacy of early psychological interventions for individuals exposed to trauma, with a particular focus on post-traumatic stress disorder (PTSD) prevention and treatment. Various approaches have been assessed across different populations, intervention settings, and providers. Prior SSIs to prevent the onset of stress related disorders and PTSD for trauma exposed adults focused on psychological debriefing and have been found to be ineffective and in some cases harmful to participants [25,26,30,36]. However, the recent evidence supporting the use of brief trauma-focused CBT to prevent the onset of trauma disorders, may suggest that adapting trauma-focused CBT into an SSI or breaking it down into multiple SSIs each targeted for a different phase of trauma recovery could potentially be a low resource and highly scalable ways to prevent stress related disorders following exposure to traumatic events in adults [20,29–36]. One of the included reviews emphasized that fewer than 10% of individuals exposed to trauma develop PTSD, underscoring the importance of targeted interventions for high-risk individuals [36]. This observation was echoed in other reviews which only saw effectiveness in improving outcomes in populations which screened positive for peritraumatic stress symptoms or acute stress disorder [29,30,33,35].

### Limitations

This commentary is intended to offer a high-level summary of the literature on SSIs and brief early interventions to prevent the onset of stress disorders in order to inform the design of an SSI for use following trauma exposure. Therefore, this was not intended to offer exhaustive summary of the literature on this topic or systematic review of the evidence. Although our screening of the included systematic reviews was comprehensive, since we did not conduct an exhaustive search of the literature, it is possible that there are additional valuable published insights that were omitted from this paper and our selection of papers could be subject to researcher bias. However, we are confident that our selection of studies is representative of the dominant themes in the literature surrounding SSIs and brief early interventions for the prevention of stress related disorders. One limitation is that the AMSTAR-2 quality assessment was conducted by a single researcher, though the research team was available to provide guidance if any questions arose or provide a second opinion while assessing the quality of the reviews. Another limitation of our review is that only English-language systematic and umbrella reviews from PubMed were included in the screening process due to the language capabilities of our research team and time constraints. The primary studies included in the reviews were not directly reviewed and we only included data reported at the review level in our analysis. Additionally, many of the included systematic reviews noted concerns regarding long-term effectiveness, methodological limitations, and variability in outcomes in the summarized trials. This highlights the need for further research to refine these interventions and determine their optimal role for delivery in community settings in response to varied forms of trauma exposure. It is clear that additional rigorous large scale randomized control trials are needed to strengthen the evidence base for both SSIs and brief early interventions to prevent the onset of stress disorders following a trauma exposure as well as establish cost-effectiveness.

## Conclusion

The findings of this commentary highlight critical considerations for designing and developing SSIs to support individuals following a trauma exposure in community settings. SSIs may represent a promising, scalable model to expand access to mental health support, especially in underserved or resource-limited settings due to their brief, cost-effective nature and growing evidence base. Recent studies suggest that incorporating active skill-building strategies, such as those derived from cognitive behavioral therapy, enhances SSI efficacy, particularly when integrated into existing care systems. These findings underscore the importance of targeted and flexible, symptom-driven approaches than can be delivered outside of clinical settings, rather than prior approaches of a blanket application of early interventions post-trauma, such as psychological debriefing. SSIs should be seen not as replacements for comprehensive mental health care, but as an early and valuable component within a continuum of care that may serve as a gateway to further support or as preventive tools for those at elevated risk. SSIs are best positioned as accessible, low-intensity supports that can complement existing care systems and extend the reach of mental health services to those who might otherwise remain underserved. This commentary lays the groundwork for the development and testing of an evidence-based SSI that extends existing mental health care services into the community and may offer timely and low intensity support to those who may otherwise never seek or receive care from traditional settings.

## Supporting information

S1 Table Assessment of included study quality using the AMSTAR-2. (XLSX)
