# Supplementary material for: Leveraging the Single-Session Intervention approach to address psychological distress following a traumatic event: Opportunities to advance early intervention for trauma
Source: PLOS Ment Health. 2026 Jun 8;3(6):e0000618. doi: 10.1371/journal.pmen.0000618 (PMC13245798; doi:10.1371/journal.pmen.0000618)
Supplement: Attachment — Submitted filename: letter to editors for revision.docx [file pmen.0000618.s002.docx]

January 26^th^ 2026

Dear Dr. Karli Montague-Cardoso,

Thank you for considering our manuscript, *“Leveraging the Single-Session Intervention approach to address psychological distress following a traumatic event: Opportunities to advance early intervention for trauma”,* for publication in PLOS Mental Health. We greatly appreciate that you will consider the review given that your journal does not typically consider unsolicited reviews. Thank you for your feedback on the type of review this manuscript should be referred to. I have updated the review and changed all references from narrative to scoping given that our review includes methods and results sections. Thank you again for considering this manuscript. We are looking forward to the feedback and comments of the peer reviewers.

Sincerely,

Kathryn O’Neill
