# Supplementary material for: Leveraging the Single-Session Intervention approach to address psychological distress following a traumatic event: Opportunities to advance early intervention for trauma
Source: PLOS Ment Health. 2026 Jun 8;3(6):e0000618. doi: 10.1371/journal.pmen.0000618 (PMC13245798; doi:10.1371/journal.pmen.0000618)
Supplement: Attachment — Submitted filename: BRIGHT Response to Reviewers_final.docx [file pmen.0000618.s003.docx]

April 6^th^ 2026

Re: Manuscript Number: PMEN-D-26-00034R1 for Resubmission to *PLOS Mental Health*

Dear Handling Editor, Dr. Karli Montague-Cardoso,

Please find enclosed our revised manuscript (PMEN-D-26-00034R1) entitled "Leveraging the Single-Session Intervention approach to address psychological distress following a traumatic event: Opportunities to advance early intervention for trauma" for resubmission to *PLOS Mental Health.*

My co-authors and I would like to thank the reviewers for their interest in our manuscript and for their constructive and supportive comments. We believe that our revised manuscript is significantly improved due to the careful review and suggestions and inquiries.

We have systematically addressed each of the reviewer’s comments and recommendations (in *Italics*). Please see our detailed response below, followed by our revisions to the text (in RED).

Thank you for being willing to consider our revised manuscript for publication. We are very grateful to the reviewers for taking the time and effort to provide such detailed feedback and comments. We believe that the revised manuscript is substantially improved. We hope that we have addressed all concerns and we will be delighted to make any additional changes as requested.

Sincerely,

Kathryn O’Neill

Anushka Patel

Avia Gray

Laura Bond

Claire Bird

Mercedes Aguerrebere

Marisol Vargas Vilugron

Denise Hernandez

Anne Marie Warren

Vikram Patel

Katherine Sanchez

John Naslund

**Corresponding Author**: Kathryn O’Neill, MPH, Department of Global Health and Social Medicine, Harvard Medical School, 641 Huntington Avenue, Boston, MA, USA. Email: katy_oneill@hms.harvard.edu

**Comments from the reviewers:**

**Reviewer 1:**

*This manuscript, “Leveraging the Single-Session Intervention approach to address psychological distress following a traumatic event: Opportunities to advance early intervention for trauma”, presents a scoping review of systematic and umbrella reviews on single-session interventions (SSIs) for mental health problems, alongside reviews of brief early psychological interventions following trauma. It then offers a conceptual synthesis and clinical/implementation considerations for how SSIs might be used as early, scalable components in a post-trauma continuum of care.*

*The topic is timely and important. The manuscript is generally well written and accessible, with coherent use of tables and a helpful figure summarising phases of trauma recovery. However, there are several issues that, in my view, require attention before the manuscript is suitable for publication as a “Research Article” based on a scoping review.*

Thank you for this thoughtful feedback. We are grateful for your thoughtful comments on our manuscript and have addressed your concerns below. We believe your feedback has strengthened our manuscript and increased its quality.

*1. The manuscript is now framed as a scoping review plus commentary, but the methodological description remains rather brief. The review does not appear to follow an established scoping review framework (e.g. JBI, PRISMA-ScR), and key elements are missing or only partially described: full search strategy, exact date ranges, inclusion/exclusion criteria beyond “<3 months and <8 sessions”, screening process (numbers screened, excluded, reasons), and whether study selection was performed in duplicate.*

*At present, a single database (PubMed) seems to have been searched. For a review of psychosocial interventions, restricting the search to PubMed alone will almost certainly have missed key systematic reviews indexed in PsycINFO, Embase or CINAHL. This is acknowledged in the limitations section only indirectly. I would strongly encourage either (a) extending the search to at least one core psychology database, or (b) explicitly acknowledging and justifying the single-database approach as a pragmatic limitation, with more emphasis on the potential for missed reviews.*

Thank you for this feedback. The intention of this review was to provide high level synthesis of the literature on both single session interventions for various mental health conditions and brief interventions of trauma and have an emphasis on a conceptual commentary of the potential of an SSI approach to address trauma responses. When we had originally submitted this manuscript, we were categorizing it as a narrative review. We relabeled this manuscript as a scoping review due to the guidance of the editor since we included a brief methods section. Upon further reflection, we have framed the article as a commentary and removed any mention of it being a ‘scoping review’ to more accurately reflect the type of manuscript that it is. Additionally, we have added to the limitations section an explicit statement that only one database was searched.

**Page 25-26:** Although our screening of the included systematic reviews was comprehensive, since we did not conduct an exhaustive search of the literature, it is possible that there are additional valuable published insights that were omitted from this paper and our selection of papers could be subject to researcher bias. However, we are confident that our selection of studies is representative of the dominant themes in the literature surrounding SSIs and brief early interventions for the prevention of stress related disorders. One limitation is that the AMSTAR-2 quality assessment was conducted by a single researcher, though the research team was available to provide guidance if any questions arose or provide a second opinion while assessing the quality of the reviews. Another limitation of our review is that only English-language systematic and umbrella reviews from PubMed were included in the screening process due to the language capabilities of our research team and time constraints. The primary studies included in the reviews were not directly reviewed and we only included data reported at the review level in our analysis.

*2. The use of AMSTAR-2 is appropriate, but the methods and implications of the ratings are under-developed. AMSTAR-2 is completed by a single reviewer. Although the team met to discuss uncertainties, this does not fully substitute for independent double-rating; this should be acknowledged explicitly.*

*The results section reports counts of reviews rated “high”, “moderate”, and “low” quality, and notes that details are in Supplementary Table 1, but the narrative synthesis does not really weight or interpret findings in light of review quality. For example, are the strongest conclusions largely driven by high-quality reviews, or are they based on reviews assessed as low quality? Please consider more explicitly linking key claims to the quality of the underlying reviews, or at minimum noting where conclusions rest primarily on low/moderate-quality evidence.*

We appreciate your constructive criticisim related to our use of the AMSTAR-2 quality assessment. We have added that the assessment was conducted by a single reviewer to the limitations section of our manuscript. The revision is included in response to your point number 7. We have also noted throughout the results section which reviews were assessed as having high, moderate or low methodological quality. We have included a few examples of this below.

**Page 11:** Both of the reviews were assessed as having high methodological quality using the AMSTAR-2 assessment (21, 24).

**Page 12:** It should be noted that one of these reviews as assessed has having moderate methodological quality (25) and the other was assess as having low methodological quality (26) using the AMSTAR-2 quality assessment.

**Page 13:** This review (28) was assessed has having moderate methodological quality using the AMSTAR-2 quality assessment.

*3. The paper currently states that the SSI reviews “examined the results of 478 clinical trials” and the trauma brief-intervention reviews “examined the results of 236 clinical trials.” Given that you include one umbrella review (Schleider et al.) plus multiple systematic reviews in related domains, there is a high likelihood of overlap in primary trials across these reviews.*

*As written, readers may infer that 478 and 236 represent unique trials, which is almost certainly not the case. Please clarify that these figures reflect the sum of trials reported across reviews, and explicitly acknowledge the likelihood of overlap (and thus that these numbers should not be interpreted as counts of unique trials). If feasible, you might indicate which reviews are subsumed within the umbrella review or have known overlap.*

Thank you for pointing this out and suggesting this clarification. We have revised the first paragraph of the results section to make this clear to the reader.

**Page 8:** We identified 6 systematic reviews and 1 umbrella review investigating the effectiveness of SSIs (Table 1), of which 3 included a meta-analysis. The sum of clinical trials reported across the reviews was 478. Due to the related nature of many of the reviews, there was likely overlap in the included trials among some of the reviews. We also identified 9 systematic reviews investigating brief early interventions to prevent the onset of PTSD and other mood disorders following trauma exposure (Table 2), of which 3 included a meta-analysis. The reviews of brief and early interventions for trauma examined the results of 236 clinical trials collectively. Since the reviews had similar research questions, it is possible that there is overlap among the included clinical trials in some of the reviews. Two reviews focusing on SSIs examined the effectiveness of an SSI for PTSD prevention and psychological debriefing. Both reviews indicated there was little to no evidence that SSI psychological debriefing was effective in preventing PTSD. Trauma-focused cognitive-behavioral therapy (CBT) consistently demonstrated the strongest evidence for PTSD prevention, particularly in individuals exhibiting early trauma symptoms (Table 2).

*4.The PFA section appropriately draws on an integrative review and highlights heterogeneity in PFA protocols. However, PFA is not included in the original search and selection process; it appears to be introduced as contextual material. This is reasonable, but should be clearly signposted as such in the Methods and Results (e.g. “In addition, we discuss PFA as an illustrative brief acute-phase intervention based on a separate integrative review, rather than as part of our primary search”).*

*You may also wish to more explicitly connect the PFA findings to your proposed SSI framework. For instance, how do PFA’s active components align with the framework and the phase-specific SSI model you propose? This would make the inclusion of PFA feel less tangential.*

This is great feedback. We have now made a change in the method section and used similar wording to what you suggest to improve clarity and transparency about the inclusion of PFA in our method (see pg. 7). We now state, “In addition, we discuss Psychological First Aid (PFA) as an illustrative case: PFA is a brief intervention designed for the acute phase (i.e., within minutes or hours of event exposure). While PFA was not explicitly part of our primary search but as it is single session in format, we lean on a separate integrative review (see Ling et al., 2025 for a detailed review).”

We have now included PFA as a potential intervention into the framework we propose in Objective 3. We include PFA in the ‘early phase’ of trauma recovery (0-2 weeks), conceptualizing it as an *acute* crisis response tool rather than a recovery-oriented intervention. We also highlight which techniques of PFA are useful in this phase. Notably, PFA is applied within minutes or hours of experiencing trauma exposure and is largely deployed in disaster settings.While PFA has a growing evidence base, it is designed for acute crisis response rather than to serve as a trauma-informed treatment with a recovery orientation.

**Page 19:** One intervention with an evidence base that can be deployed for crisis management in this phase is PFA. We conceptualize PFA as an immediate *acute* crisis response tool rather than a recovery-oriented intervention. While the implementation of PFA has drifted in practice (Ling et al., 2025), its core techniques of anxiety management, linkage to resources, and problem-solving remain valuable tools for stabilizing a survivor in the minutes or hours after a traumatic event.

*5. The manuscript argues that SSIs could be especially valuable in underserved community settings, yet the included reviews and the primary studies they summarise are largely from high-income countries. A short subsection explicitly addressing issues of global applicability, cultural adaptation, and the paucity of evidence from low- and middle-income settings (including community and humanitarian contexts) would strengthen the paper, particularly for a journal with a global mental health remit.*

We agree with your point entirely; thank you for this feedback. We now make this argument more concrete by including a short subsection, as you suggest, to preface design consideraitons in Objective 3.

**Page 18:** Almost all the of SSIs reviewed in our study were designed and deployed in high-income and westernized cultural settings. Ample evidence now suggests that the clinical presentation of PTSD varies by cultural setting (44, 45). Yet, the lack of rigorous and systematic evaluations on brief interventions in general and SSIs in particular from low-and-middle-income countries is rather striking. Currently, there are very few SSIs designed and deployed for high-risk settings where humanitarian and natural disasters occur. Given the brevity and ease of implementing SSIs once providers are trained and that LMIC shoulder the lion’s share of natural disasters and humanitarian emergencies (46), this underscores a missed opportunity. In this next section, we provide clinical and implementation considerations to design SSIs, and these principles should be applied for underserved communities who have limited access to treatment resources.

*6. The abstract and introduction describe this as a “commentary and scoping review.” PLOS classifies it as a Research Article. It would help the reader if you clearly articulate in the introduction that this is a review of reviews plus conceptual/practice implications, rather than a primary empirical scoping review.*

We appreciate this feedback and the need for clarification. We have changed the langague to describe this as a commentary, revised a sentence describing the manuscript in the introduction and included the revised version below for your reference.

**Page 6:** Therefore, by reviewing and reflecting on the existing literature in this commentary examining published systematic and umbrella reviews, we offer insights for guiding the development and use of SSIs for early intervention in response to both interpersonal and community trauma exposure in a conceptual commentary on implications for practice.

*7.Limitations paragraph – Please explicitly state that (1) only English-language systematic/umbrella reviews indexed in PubMed were considered; (2) primary studies were not directly reviewed; and (3) the AMSTAR-2 appraisal was conducted by a single reviewer. These are already alluded to in various ways, but a concise list of key methodological limitations would be useful.*

Thank you for this suggestion. We have added these additional considerations to the limitations section of our manuscript. You may see the additional information we have included to address this comment below.

**Page 25-26:** One limitation is that the AMSTAR-2 quality assessment was conducted by a single researcher, though the research team was available to provide guidance if any questions arose or provide a second opinion while assessing the quality of the reviews. Another limitation of our review is that only English-language systematic and umbrella reviews from PubMed were included in the screening process due to the language capabilities of our research team and time constraints. The primary studies included in the reviews were not directly reviewed and we only included data reported at the review level in our analysis.

**Reviewer 2:**

*The authors have significantly improved the manuscript (R1), particularly in terms of structure and conceptual clarity. The revised version addresses an important gap in the literature on trauma-related interventions and aligns well with the aims of PLOS Mental Health. Overall, the transition to a scoping review format has strengthened the manuscript. While the manuscript is close to being suitable for publication, a few minor refinements would further enhance its scientific rigor and balance.*

*The revised manuscript (PMEN-D-26-00034R1) provides a valuable and timely perspective on the use of Single-Session Interventions (SSIs) for trauma-related distress. The scoping review approach improves transparency and organization. The remaining comments are minor in nature and can be addressed without substantial restructuring.*

Thank you for your comments and feedback. We appreciate you taking the time for a thorough review of this paper and your appreciation of the timeliness of this manuscript.

*1.Throughout the manuscript, particularly in the Abstract and Discussion sections, causal language should be further tempered. Given the scoping review design, phrases such as “addressing” or “reducing” distress would benefit from more cautious wording (e.g., “potential to mitigate,” “may support,” or “preliminary evidence suggests”).*

Thank you for this feedback. We have edited the manuscript to further temper any causal language that has been used. Please see some examples below of statements that have been revised.

**Page 2:** Overall, the SSI approach may offer promise for improving mental health outcomes after traumatic experiences.

**Page 22:** The summarized body of research on SSIs suggests their potential to mitigate various mental health conditions, though their effectiveness varies depending on the disorder, population, and treatment approach.

**Page 23:** Although SSIs and early interventions may offer promising short-term benefits, their long-term effectiveness remains uncertain, emphasizing the need for further research on targeted approaches and implementation strategies (17, 18).

**Page 24:** Integrating SSIs into existing care systems as a first line treatment may have potential to bridge gaps in support systems.

*2.The AMSTAR-2 quality assessment was conducted by a single researcher. While this is understandable given the scope of the review, explicitly acknowledging this as a limitation in the Discussion would further strengthen methodological transparency and address potential bias.*

Thank you for this suggestion. We agree that this should be noted as methodological limitation of our manuscript. We have addressed this and added a statement in response to Review 1’s comment #7.

*3. Please ensure that all remaining references to “narrative review” are removed and consistently replaced with “scoping review” throughout the manuscript, including any section headers or discussion passages.*

Thank you for this suggestion. We have double checked the manuscript to ensure that all references to “narrative review” have been replaced with “scoping review”.

*4. A brief acknowledgment in the Discussion that the effectiveness and suitability of SSIs may vary depending on trauma type (e.g., acute vs. chronic, interpersonal vs. non-interpersonal) would improve conceptual nuance.*

Thank you for pointing out this nuance. We have now acknowledged that effects are likely to vary by trauma type.

**Page 23:** Similarly, it is crucial to consider the clinical nuance of the index traumatic event and individual differences in response when deciding to deploy an SSI. For example, prolonged childhood abuse and neglect tends to elicit a complex PTSD response compared to single-event incidents and accidental events (e.g., natural disaster or car accident). Similarly, certain events – such as drug-facilitated sexual assault – can elicit high levels of shame and impair trauma memory to create dissociative responses whereas other traumatic exposures do not come with this presentation (69, 70). Ultimately, the range of traumatic events and individual responses – which are heterogenous in clinical presentation – are crucial nuances to consider when developing and deploying an SSI.

*5. It would be beneficial to briefly address the potential for iatrogenic effects in early trauma interventions and to note the importance of appropriate safeguards and screening within the proposed SSI framework.*

We now include this caveat into objective three (pg 21) and the discussion (pg 23-24).
