# Supplementary material for: Leveraging the Single-Session Intervention approach to address psychological distress following a traumatic event: Opportunities to advance early intervention for trauma
Source: PLOS Ment Health. 2026 Jun 8;3(6):e0000618. doi: 10.1371/journal.pmen.0000618 (PMC13245798; doi:10.1371/journal.pmen.0000618)
Supplement: Peer review history

# Editor report: Decision Letter 0

**DOI:** 10.1371/journal.pmen.0000618.r001

**Authors:** Karli Montague-Cardoso

23 Jan 2026

PMEN-D-26-00034

Leveraging the Single-Session Intervention approach to address psychological distress following a traumatic event: Opportunities to advance early intervention for trauma

PLOS Mental Health

Dear Dr. O'Neill,

Thank you for submitting your Review to PLOS Mental Health. We do not usually consider unsolicited Reviews but I think your Review addresses a very important topic and so I would be happy to consider this and send it out to review (all of our Reviews and Essays are peer reviewed). Before I do that however, I would like to request a minor revision/ask for clarification. You have stated that the piece is a Narrative Review. However at present, given the way it is presented, with a clear lay out of 'methods' and 'results', it could be viewed as a Scoping Review. The differentiation is important as we treat Scoping Reviews as Research Articles due to their intention to produce new findings (and therefore they have an APC, unlike Narrative Reviews). If you intend to keep the piece as it is, then upon resubmission it would need to be classified as a Research Article and referred to as a Scoping Review. If however, you did not intend to go down this route, please revise the piece so that it is not presented as a Scoping Review. A Narrative Review should not refer to methods or results specifically and instead should consist of an Introduction before you then move straight into a synthesis of the literature. If you have any questions or would like me to take a look before you resubmit, please reach out to me directly at kmontague-cardoso@plos.org.

Please submit your revised manuscript by Mar 07 2026 11:59PM. If you will need more time than this to complete your revisions, please reply to this message or contact the journal office at mentalhealth@plos.org. When you're ready to submit your revision, log on to https://www.editorialmanager.com/pmen/ and select the 'Submissions Needing Revision' folder to locate your manuscript file.

Please include the following items when submitting your revised manuscript:

- A letter that responds to each point raised by the editor and reviewer(s). You should upload this letter as a separate file labeled 'Response to Reviewers'.
- A marked-up copy of your manuscript that highlights changes made to the original version. You should upload this as a separate file labeled 'Revised Manuscript with Track Changes'.
- An unmarked version of your revised paper without tracked changes. You should upload this as a separate file labeled 'Manuscript'.

Guidelines for resubmitting your figure files are available below the reviewer comments at the end of this letter.

We look forward to receiving your revised manuscript.

Kind regards,

Karli Montague-Cardoso

Staff Editor

PLOS Mental Health

Journal Requirements:

If the reviewer comments include a recommendation to cite specific previously published works, please review and evaluate these publications to determine whether they are relevant and should be cited. There is no requirement to cite these works unless the editor has indicated otherwise.

Please review your reference list to ensure that it is complete and correct. If you have cited papers that have been retracted, please include the rationale for doing so in the manuscript text, or remove these references and replace them with relevant current references. Any changes to the reference list should be mentioned in the rebuttal letter that accompanies your revised manuscript. If you need to cite a retracted article, indicate the article’s retracted status in the References list and also include a citation and full reference for the retraction notice.

Additional Editor Comments (if provided):

[Note: HTML markup is below. Please do not edit.]

Reviewers' comments:

[NOTE: If reviewer comments were submitted as an attachment file, they will be attached to this email and accessible via the submission site. Please log into your account, locate the manuscript record, and check for the action link "View Attachments". If this link does not appear, there are no attachment files.]

-->Figure Resubmissions:

While revising your submission, we strongly recommend that you use PLOS’s NAAS tool (https://ngplosjournals.pagemajik.ai/artanalysis) to test your figure files. NAAS can convert your figure files to the TIFF file type and meet basic requirements (such as print size, resolution), or provide you with a report on issues that do not meet our requirements and that NAAS cannot fix.

After uploading your figures to PLOS’s NAAS tool - https://ngplosjournals.pagemajik.ai/artanalysis, NAAS will process the files provided and display the results in the "Uploaded Files" section of the page as the processing is complete. If the uploaded figures meet our requirements (or NAAS is able to fix the files to meet our requirements), the figure will be marked as "fixed" above. If NAAS is unable to fix the files, a red "failed" label will appear above. When NAAS has confirmed that the figure files meet our requirements, please download the file via the download option, and include these NAAS processed figure files when submitting your revised manuscript.

# Author comment: Author response to Decision Letter 1

**DOI:** 10.1371/journal.pmen.0000618.r002

26 Jan 2026

Attachment Submitted filename: letter to editors for revision.docx

# Peer review: Decision Letter 1

**DOI:** 10.1371/journal.pmen.0000618.r003

**Authors:** Karli Montague-Cardoso

9 Feb 2026

PMEN-D-26-00034R1

Leveraging the Single-Session Intervention approach to address psychological distress following a traumatic event: Opportunities to advance early intervention for trauma

PLOS Mental Health

Dear Dr. O'Neill,

Thank you for submitting your manuscript to PLOS Mental Health. After careful consideration of the reviewer reports, we feel that it has merit but does not fully meet PLOS Mental Health’s publication criteria as it currently stands. Therefore, we invite you to submit a revised version of the manuscript that addresses the points raised during the review process. You can find all of the comments raised at the end of this email.

Please submit your revised manuscript by Mar 26 2026 11:59PM. If you will need more time than this to complete your revisions, please reply to this message or contact the journal office at mentalhealth@plos.org. When you're ready to submit your revision, log on to https://www.editorialmanager.com/pmen/ and select the 'Submissions Needing Revision' folder to locate your manuscript file.

Please include the following items when submitting your revised manuscript:

- A letter that responds to each point raised by the editor and reviewer(s). You should upload this letter as a separate file labeled 'Response to Reviewers'.
- A marked-up copy of your manuscript that highlights changes made to the original version. You should upload this as a separate file labeled 'Revised Manuscript with Track Changes'.
- An unmarked version of your revised paper without tracked changes. You should upload this as a separate file labeled 'Manuscript'.

Guidelines for resubmitting your figure files are available below the reviewer comments at the end of this letter.

We look forward to receiving your revised manuscript.

Kind regards,

Karli Montague-Cardoso

Staff Editor

PLOS Mental Health

Journal Requirements:

If the reviewer comments include a recommendation to cite specific previously published works, please review and evaluate these publications to determine whether they are relevant and should be cited. There is no requirement to cite these works unless the editor has indicated otherwise.

Additional Editor Comments (if provided):

[Note: HTML markup is below. Please do not edit.]

Reviewers' comments:

Reviewer's Responses to Questions

**Comments to the Author**

1. If the authors have adequately addressed your comments raised in a previous round of review and you feel that this manuscript is now acceptable for publication, you may indicate that here to bypass the “Comments to the Author” section, enter your conflict of interest statement in the “Confidential to Editor” section, and submit your "Accept" recommendation.

Reviewer #1: (No Response)

Reviewer #2: All comments have been addressed

**********

2. Does this manuscript meet PLOS Mental Health’s publication criteria? Is the manuscript technically sound, and do the data support the conclusions? The manuscript must describe methodologically and ethically rigorous research with conclusions that are appropriately drawn based on the data presented.

Reviewer #1: Yes

Reviewer #2: Yes

**********

3. Has the statistical analysis been performed appropriately and rigorously?

Reviewer #1: Yes

Reviewer #2: Yes

**********

4. Have the authors made all data underlying the findings in their manuscript fully available (please refer to the Data Availability Statement at the start of the manuscript PDF file)?

The PLOS Data policy requires authors to make all data underlying the findings described in their manuscript fully available without restriction, with rare exception. The data should be provided as part of the manuscript or its supporting information, or deposited to a public repository. For example, in addition to summary statistics, the data points behind means, medians and variance measures should be available. If there are restrictions on publicly sharing data—e.g. participant privacy or use of data from a third party—those must be specified.

Reviewer #1: Yes

Reviewer #2: Yes

**********

5. Is the manuscript presented in an intelligible fashion and written in standard English?

PLOS Mental Health does not copyedit accepted manuscripts, so the language in submitted articles must be clear, correct, and unambiguous. Any typographical or grammatical errors should be corrected at revision, so please note any specific errors here.

Reviewer #1: Yes

Reviewer #2: Yes

**********

6. Review Comments to the Author

Please use the space provided to explain your answers to the questions above. You may also include additional comments for the author, including concerns about dual publication, research ethics, or publication ethics. (Please upload your review as an attachment if it exceeds 20,000 characters)

Reviewer #1: This manuscript, “Leveraging the Single-Session Intervention approach to address psychological distress following a traumatic event: Opportunities to advance early intervention for trauma”, presents a scoping review of systematic and umbrella reviews on single-session interventions (SSIs) for mental health problems, alongside reviews of brief early psychological interventions following trauma. It then offers a conceptual synthesis and clinical/implementation considerations for how SSIs might be used as early, scalable components in a post-trauma continuum of care.

The topic is timely and important. The manuscript is generally well written and accessible, with coherent use of tables and a helpful figure summarising phases of trauma recovery. However, there are several issues that, in my view, require attention before the manuscript is suitable for publication as a “Research Article” based on a scoping review.

Overall recommendation: major revision.

1. The manuscript is now framed as a scoping review plus commentary, but the methodological description remains rather brief. The review does not appear to follow an established scoping review framework (e.g. JBI, PRISMA-ScR), and key elements are missing or only partially described: full search strategy, exact date ranges, inclusion/exclusion criteria beyond “<3 months and <8 sessions”, screening process (numbers screened, excluded, reasons), and whether study selection was performed in duplicate.

At present, a single database (PubMed) seems to have been searched. For a review of psychosocial interventions, restricting the search to PubMed alone will almost certainly have missed key systematic reviews indexed in PsycINFO, Embase or CINAHL. This is acknowledged in the limitations section only indirectly. I would strongly encourage either (a) extending the search to at least one core psychology database, or (b) explicitly acknowledging and justifying the single-database approach as a pragmatic limitation, with more emphasis on the potential for missed reviews.

2. The use of AMSTAR-2 is appropriate, but the methods and implications of the ratings are under-developed. AMSTAR-2 is completed by a single reviewer. Although the team met to discuss uncertainties, this does not fully substitute for independent double-rating; this should be acknowledged explicitly.

The results section reports counts of reviews rated “high”, “moderate”, and “low” quality, and notes that details are in Supplementary Table 1, but the narrative synthesis does not really weight or interpret findings in light of review quality. For example, are the strongest conclusions largely driven by high-quality reviews, or are they based on reviews assessed as low quality? Please consider more explicitly linking key claims to the quality of the underlying reviews, or at minimum noting where conclusions rest primarily on low/moderate-quality evidence.

3. The paper currently states that the SSI reviews “examined the results of 478 clinical trials” and the trauma brief-intervention reviews “examined the results of 236 clinical trials.” Given that you include one umbrella review (Schleider et al.) plus multiple systematic reviews in related domains, there is a high likelihood of overlap in primary trials across these reviews.

As written, readers may infer that 478 and 236 represent unique trials, which is almost certainly not the case. Please clarify that these figures reflect the sum of trials reported across reviews, and explicitly acknowledge the likelihood of overlap (and thus that these numbers should not be interpreted as counts of unique trials). If feasible, you might indicate which reviews are subsumed within the umbrella review or have known overlap.

4.The PFA section appropriately draws on an integrative review and highlights heterogeneity in PFA protocols. However, PFA is not included in the original search and selection process; it appears to be introduced as contextual material. This is reasonable, but should be clearly signposted as such in the Methods and Results (e.g. “In addition, we discuss PFA as an illustrative brief acute-phase intervention based on a separate integrative review, rather than as part of our primary search”).

You may also wish to more explicitly connect the PFA findings to your proposed SSI framework. For instance, how do PFA’s active components align with the framework and the phase-specific SSI model you propose? This would make the inclusion of PFA feel less tangential.

5. The manuscript argues that SSIs could be especially valuable in underserved community settings, yet the included reviews and the primary studies they summarise are largely from high-income countries. A short subsection explicitly addressing issues of global applicability, cultural adaptation, and the paucity of evidence from low- and middle-income settings (including community and humanitarian contexts) would strengthen the paper, particularly for a journal with a global mental health remit.

6. The abstract and introduction describe this as a “commentary and scoping review.” PLOS classifies it as a Research Article. It would help the reader if you clearly articulate in the introduction that this is a review of reviews plus conceptual/practice implications, rather than a primary empirical scoping review.

7.Limitations paragraph – Please explicitly state that (1) only English-language systematic/umbrella reviews indexed in PubMed were considered; (2) primary studies were not directly reviewed; and (3) the AMSTAR-2 appraisal was conducted by a single reviewer. These are already alluded to in various ways, but a concise list of key methodological limitations would be useful.

Reviewer #2: The authors have significantly improved the manuscript (R1), particularly in terms of structure and conceptual clarity. The revised version addresses an important gap in the literature on trauma-related interventions and aligns well with the aims of PLOS Mental Health. Overall, the transition to a scoping review format has strengthened the manuscript. While the manuscript is close to being suitable for publication, a few minor refinements would further enhance its scientific rigor and balance.

General Comments

The revised manuscript (PMEN-D-26-00034R1) provides a valuable and timely perspective on the use of Single-Session Interventions (SSIs) for trauma-related distress. The scoping review approach improves transparency and organization. The remaining comments are minor in nature and can be addressed without substantial restructuring.

Specific Points for Revision

1. Throughout the manuscript, particularly in the Abstract and Discussion sections, causal language should be further tempered. Given the scoping review design, phrases such as “addressing” or “reducing” distress would benefit from more cautious wording (e.g., “potential to mitigate,” “may support,” or “preliminary evidence suggests”).

2.The AMSTAR-2 quality assessment was conducted by a single researcher. While this is understandable given the scope of the review, explicitly acknowledging this as a limitation in the Discussion would further strengthen methodological transparency and address potential bias.

3. Please ensure that all remaining references to “narrative review” are removed and consistently replaced with “scoping review” throughout the manuscript, including any section headers or discussion passages.

4. A brief acknowledgment in the Discussion that the effectiveness and suitability of SSIs may vary depending on trauma type (e.g., acute vs. chronic, interpersonal vs. non-interpersonal) would improve conceptual nuance.

5. It would be beneficial to briefly address the potential for iatrogenic effects in early trauma interventions and to note the importance of appropriate safeguards and screening within the proposed SSI framework.

Addressing these minor points would further strengthen the manuscript and enhance its contribution to the field.

**********

7. PLOS authors have the option to publish the peer review history of their article (what does this mean?). If published, this will include your full peer review and any attached files.

**Do you want your identity to be public for this peer review?** If you choose “no”, your identity will remain anonymous but your review may still be made public.

For information about this choice, including consent withdrawal, please see our Privacy Policy.

Reviewer #1: **Yes:** Ling Wang

Reviewer #2: **Yes:** Cigdem Kinik

**********

[NOTE: If reviewer comments were submitted as an attachment file, they will be attached to this email and accessible via the submission site. Please log into your account, locate the manuscript record, and check for the action link "View Attachments". If this link does not appear, there are no attachment files.]

-->Figure Resubmissions:

While revising your submission, we strongly recommend that you use PLOS’s NAAS tool (https://ngplosjournals.pagemajik.ai/artanalysis) to test your figure files. NAAS can convert your figure files to the TIFF file type and meet basic requirements (such as print size, resolution), or provide you with a report on issues that do not meet our requirements and that NAAS cannot fix.

After uploading your figures to PLOS’s NAAS tool - https://ngplosjournals.pagemajik.ai/artanalysis, NAAS will process the files provided and display the results in the "Uploaded Files" section of the page as the processing is complete. If the uploaded figures meet our requirements (or NAAS is able to fix the files to meet our requirements), the figure will be marked as "fixed" above. If NAAS is unable to fix the files, a red "failed" label will appear above. When NAAS has confirmed that the figure files meet our requirements, please download the file via the download option, and include these NAAS processed figure files when submitting your revised manuscript.

# Author comment: Author response to Decision Letter 2

**DOI:** 10.1371/journal.pmen.0000618.r004

6 Apr 2026

Attachment Submitted filename: BRIGHT Response to Reviewers_final.docx

# Peer review: Decision Letter 2

**DOI:** 10.1371/journal.pmen.0000618.r005

**Authors:** Karli Montague-Cardoso

28 Apr 2026

Leveraging the Single-Session Intervention approach to address psychological distress following a traumatic event: Opportunities to advance early intervention for trauma

PMEN-D-26-00034R2

Dear Ms. O'Neill,

We are pleased to inform you that your manuscript 'Leveraging the Single-Session Intervention approach to address psychological distress following a traumatic event: Opportunities to advance early intervention for trauma' has been provisionally accepted for publication in PLOS Mental Health.

Before your manuscript can be formally accepted you will need to complete some formatting changes, which you will receive in a follow up email. A member of our team will be in touch with a set of requests.

Please note that your manuscript will not be scheduled for publication until you have made the required changes, so a swift response is appreciated.

IMPORTANT: The editorial review process is now complete. PLOS will only permit corrections to spelling, formatting or significant scientific errors from this point onwards. Requests for major changes, or any which affect the scientific understanding of your work, will cause delays to the publication date of your manuscript.

If your institution or institutions have a press office, please notify them about your upcoming paper to help maximize its impact. If they'll be preparing press materials, please inform our press team as soon as possible -- no later than 48 hours after receiving the formal acceptance. Your manuscript will remain under strict press embargo until 2 pm Eastern Time on the date of publication. For more information, please contact mentalhealth@plos.org.

Thank you again for supporting Open Access publishing; we are looking forward to publishing your work in PLOS Mental Health.

Best regards,

Karli Montague-Cardoso

Staff Editor

PLOS Mental Health

***********************************************************

Reviewer Comments (if any, and for reference):

Reviewer's Responses to Questions

**Comments to the Author**

1. If the authors have adequately addressed your comments raised in a previous round of review and you feel that this manuscript is now acceptable for publication, you may indicate that here to bypass the “Comments to the Author” section, enter your conflict of interest statement in the “Confidential to Editor” section, and submit your "Accept" recommendation.

Reviewer #1: All comments have been addressed

**********

2. Does this manuscript meet PLOS Mental Health’s publication criteria? Is the manuscript technically sound, and do the data support the conclusions? The manuscript must describe methodologically and ethically rigorous research with conclusions that are appropriately drawn based on the data presented.

Reviewer #1: Yes

**********

3. Has the statistical analysis been performed appropriately and rigorously?

Reviewer #1: Yes

**********

4. Have the authors made all data underlying the findings in their manuscript fully available (please refer to the Data Availability Statement at the start of the manuscript PDF file)?

The PLOS Data policy requires authors to make all data underlying the findings described in their manuscript fully available without restriction, with rare exception. The data should be provided as part of the manuscript or its supporting information, or deposited to a public repository. For example, in addition to summary statistics, the data points behind means, medians and variance measures should be available. If there are restrictions on publicly sharing data—e.g. participant privacy or use of data from a third party—those must be specified.

Reviewer #1: Yes

**********

5. Is the manuscript presented in an intelligible fashion and written in standard English?

PLOS Mental Health does not copyedit accepted manuscripts, so the language in submitted articles must be clear, correct, and unambiguous. Any typographical or grammatical errors should be corrected at revision, so please note any specific errors here.

Reviewer #1: Yes

**********

6. Review Comments to the Author

Please use the space provided to explain your answers to the questions above. You may also include additional comments for the author, including concerns about dual publication, research ethics, or publication ethics. (Please upload your review as an attachment if it exceeds 20,000 characters)

Reviewer #1: The authors have addressed my previous comments in a satisfactory manner. The revised manuscript is now more accurately positioned as a commentary and review of reviews rather than a full scoping review, which better matches the methodological approach taken. The authors have also strengthened the methodological transparency by clarifying the use of PubMed only, acknowledging the inclusion of English-language systematic/umbrella reviews, noting that primary studies were not directly reviewed, and stating that the AMSTAR-2 assessment was conducted by a single reviewer.

**********

7. PLOS authors have the option to publish the peer review history of their article (what does this mean?). If published, this will include your full peer review and any attached files.

**Do you want your identity to be public for this peer review?** If you choose “no”, your identity will remain anonymous but your review may still be made public.

For information about this choice, including consent withdrawal, please see our Privacy Policy.

Reviewer #1: **Yes:** Ling Wang

**********
